# *SCN9A* rs6746030 Polymorphism and Pain Perception in Combat Athletes and Non-Athletes

**DOI:** 10.3390/genes14030733

**Published:** 2023-03-16

**Authors:** Katarzyna Leźnicka, Maciej Pawlak, Marek Sawczuk, Agata Gasiorowska, Agata Leońska-Duniec

**Affiliations:** 1 Faculty of Physical Education, Gdansk University of Physical Education and Sport, 80-336 Gdansk, Poland; 2 Department of Physiology and Biochemistry, Poznan University of Physical Education, 61-871 Poznan, Poland; 3 Institute of Physical Culture Sciences, University of Szczecin, 70-453 Szczecin, Poland; 4 Faculty of Psychology in Wroclaw, SWPS University of Social Sciences and Humanities, Ostrowskiego 30b, 54-238 Wroclaw, Poland

**Keywords:** genetics, pain threshold, pain tolerance, combat athletes, *SCN9A*, polymorphism

## Abstract

One of the genes associated with pain perception is *SCN9A*, which encodes an α-subunit of the voltage gated sodium channel, NaV1.7, a crucial player in peripheral pain sensation. It has been suggested that a common missense polymorphism within *SCN9A* (rs6746030; G>A; R1150W) may affect nociception in the general population, but its effects of pain perception in athletes remain unknown. Therefore, the aim of the study was to investigate the association between a polymorphism within *SCN9A* (rs6746030) and pain perception (pain threshold and pain tolerance) in the group of combat athletes (*n* = 214) and students (*n* = 92) who did not participate in sports at a professional level. Genotyping was carried out using TaqMan Real-Time PCR method. No significant differences were found between the *SCN9A* genotype distributions with respect to the pain threshold. However, the probability of having a high pain threshold was higher in the combat sports group than in the control group. The probability of having a decreased pain tolerance was higher in the carriers of the GA and AA genotype than in the homozygotes of the GG genotype. Moreover, the possibility of having a high pain threshold was higher in the combat athlete group than in the control group. The results of our study suggest that the *SCN9A* rs6746030 polymorphism may affect pain perception. However, the additional effect of the experimental group may suggest that pain tolerance is significantly modulated by other factors, such as the systematic exposure of the athletes’ bodies to short-term high-intensity stimuli during training sessions.

## 1. Introduction

Pain is defined as an unpleasant sensory and emotional experience associated with actual or potential tissue damage or described in terms of such damage [1]. A negative emotional attitude towards pain occurs at every stage of life, beginning already in fetal life and manifested in neuropsychological, hormonal, and behavioral reactions to pain and non-pain stimuli [2].

By recognizing external and internal threats to the body, pain performs an extremely important function, especially task in the field of information, warning, and protection, which are crucial for the survival of each human being. Pain is admittedly considered as a subjective experience, but its quantitative and qualitative dimension is continuously modulated and modified by numerous factors such as social, psychological, religious, cultural, and personality factors, as well as physical activity [2].

Scientific research by Heneweer et al. [3] has shown that both too little and too much physical activity predisposes patients to the onset of pain. The constant or regular influx of nociceptive stimuli leads to changes in the pain processing system, including peripheral and central sensitization processes and activation of antinociceptive mechanisms. Increased pain sensitivity is associated with numerous pain disorders and is considered one of the features of central sensitization of the nervous system [4]. The phenomenon of pain is of particular interest to healthy individuals exposed to physical exertion, especially in athletes who accept pain as an integral part of their daily experience. Many hours of intensive training and frequent participation in competitions (fights) make these athletes a group that is very often exposed to intense pain [5]. On the other hand, according to theoretical studies and empirical reports, physical activity is considered one of the most important elements of pain prevention and treatment. The significant differentiation of pain response, both in patients and in physically active individuals and competitive athletes, confirms the multifactorial nature of this phenomenon [6]. One of the underlying mechanisms for individual differences in pain perception is genetic susceptibility. Multiple genes, derived from human and animal studies, have been shown to be important in modulating pain perception [7,8,9]. Literature research conducted by Wistrom et al. [7] allowed for the identification of 242 genes linked to pain-associated behaviors. Due to the function of the pain gene products, they were divided into six functional groups: (1) voltage-gated and ligand-gated ion channels (e.g., sodium channel NaV1.7—*SCN9A*); (2) G protein-coupled receptors—GPCRs (e.g., delta-opioid receptor—*OPRD1*); (3) neuropeptides, neurotransmitters, and neurotrophins (e.g., tachykinin 1—*TAC1*); (4) growth factors, hormones, and cytokines (e.g., interleukin 10—*IL-10*); (5) enzymes and enzyme-linked receptors (e.g., mitogen-activated protein kinase 1—*MAPK1*); and (6) transcriptional and translational control and mRNA processing (e.g., PR domain-containing protein 12—*PRDM12*) [7,9,10,11,12].

Recent studies have revealed that variations in voltage-gated sodium channel genes are key players in peripheral pain processing [6,7,8]. For example, inactivating mutations in *SCN9A* result in congenital insensitivity to pain, whereas gain-of-function mutations generate different pain syndromes such as inherited erythromelalgia, paroxysmal extreme pain disorder, and small-fiber neuropathy [13]. This gene has been extensively linked to human and animal pain perception [13,14,15,16]. Yet, despite over a decade of research into *SCN9A*, to the best of our knowledge, their relationship with sport skills is almost unknown. A small number of studies on association between the *SCN9A* genotypes and pain perception in athletes led us to interest this important issue. The management of pain by athletes and the pursuit of pain control is an integral part of sports, but it is also one of the most important skills in combat athletes. Because athletes are systematically exposed to brief periods of intense pain during training or competition, they must learn to manage pain effectively. Therefore, knowledge of the genetic determinants of the physiological and psychological aspects of pain in athletes, especially martial artists, can potentially be used in the selection of new sports talent and provides an additional source of detailed information useful in personalizing training methods and more effectively managing the sports careers of trained athletes [5].

Sodium channels are integral membrane proteins and consist of a large α-subunit that forms the voltage-sensitive and ion-selective pore and a small β-helper subunit(s) that can regulate the kinetics and voltage dependence of channel gating [17]. To date, nine isoforms of the α-subunit of the sodium channel (NaV1.1–NaV1.9) have been described, but NaV1.7 appears to play a critical role in pain perception [14,18,19]. The role of these channels is central due to the generation and repetitive firing properties of the different neurons [20].

*SCN9A* is a 113.5 kb gene located on the long arm of chromosome 2 (2q24.3) and consisting of 26 exons. The protein consists of 1977 amino acids organized into four domains, each with six transmembrane segments, and is expressed at high density in peripheral sensory neurons, especially in nociceptive neurons [19]. Mutations in *SCN9A* that alter NaV1.7 function and are associated with various channelopathies leading to electrical hyperactivity of sensory neurons in the dorsal root and low reactivity of neurons in the sympathetic ganglia have been shown to be causative factors in various human pain perceptions [21]. It has been suggested that a common missense polymorphism in exon 18 of *SCN9A* (rs6746030; G>A; R1150W) may influence nociception in the general population. The minor A allele has been associated with increased NaV1.7 channel activity, resulting in increased pain ratings and lower pain threshold compared with the major G allele [15].

To date, there is no gold standard for pain assessment. This is a consequence of the specificity of this multidimensional sensory phenomenon, in which peripherally registered tissue-damaging stimuli are processed in the brain and supplemented by a subjective aspect. Most often, the perceived pain is still the effect of the current psychological state, social relations, well-being, etc. In such a situation, the use of experimental pain measurements, where it should be assumed that the experimental pain response is a stable trait, provides an opportunity to investigate the use of a genetic model to predict individual differences in experimental pain in healthy young people. In the current study, we significantly reduced the number of variables and aimed to evaluate the association between *SCN9A* genotypes and pain perception (pain threshold and pain tolerance) in the group of combat athletes and men who do not play sports professionally. Firstly, following the literature, we expected that the proportion of participants with low pain sensitivity (high pain threshold and high pain tolerance) would be higher among combat athletes than among men who do not play sports professionally. Secondly, since the A allele seems to confer enhanced pain sensitivity, we hypothesized that the chance of having low pain sensitivity will be higher for the group of participants with the GG homozygous genotype in comparison to the AA homozygous genotype and the GA heterozygous genotype. Thirdly, we had no specific expectations concerning whether these two variables would interact with each other, that is, whether the effect of physical activity would depend on the *SCN9A* gene.

## 2. Materials and Methods

### 2.1. Ethics Statement

The Pomeranian Medical University Ethics Committee (Szczecin, Poland) approved the study (no. 09/KB/V/2013). The investigation protocols were conducted ethically ac-cording to the World Medical Association Declaration of Helsinki and to the Strengthening the Reporting of Genetic Association studies statement (STREGA). The participants were informed of the risks and benefits of the experimental protocols, and a written consent form was completed by each participant. All personal information and results were anonymous.

### 2.2. Participants

For the study, 306 healthy Caucasian men aged 18 to 32 years were recruited. The experimental group included 214 combat athletes (24.7 ± 6.6), and the control group consisted of 92 students (21.2 ± 1.8) who did not practice sports professionally. The combat athletes group consisted of athletes who had at least 5 years of experience in disciplines such as boxing (*n* = 101), karate (*n* = 85), and mixed martial arts (*n* = 28).

### 2.3. Pressure Pain Test (PPT)

Tissue pressure sensitivity was measured using an algometer from Quirumed (Spain). The device is a force gauge ranging from 0 to 10 kg with a disc-shaped tip covered with a rubber sheath with an area of 1 cm^2^. Pain threshold and pain tolerance were measured.

Pain threshold (PPT1) is the lowest intensity of a particular stimulus—sound, heat, touch—at which a person begins to feel pain.

Pain tolerance (PPT2) is the maximum amount of pain a person can tolerate.

The results obtained by all subjects were divided into two categories below or above 10 kg. The recording of the result in the category below and above 10.1 kg/cm^2^ resulted from the measurement limits of the device.

Before starting the measurement of pressure sensitivity, each participant was informed about the purpose of the study and received detailed instructions on how to perform the study and how to behave. Three test measurements were then performed to demonstrate pain-inducing pressure.

The study was performed in a sitting position on the dominant extremity. Participants were asked to place their hand on the table. Initially, the researcher determined the contact site by palpation, then placed the pressure head on the back of the hand between the thumb and forefinger at a 90° angle and applied pressure with increasing force at a rate of 100 g/s to the selected site. The measurement results were visible only to the person conducting the test.

### 2.4. Genotyping

Genomic DNA was extracted from the buccal cells by a Genomic Micro AX SWAB Gravity (A&A Biotechnology, Poland) according to the producer’s protocol. An allelic discrimination assay on a C1000 Touch Thermal Cycler (Bio-Rad, Feldkirchen, Germany) instrument with TaqMan^®^ probes was applied. To discriminate the *SCN9A* alleles, TaqMan^®^ Pre-Designed SNP Genotyping Assays (Applied Biosystems, Waltham, MA, USA) (assay ID: C__29330435_10), consisting of fluorescently labelled (FAM and VIC) minor groove binder (MGB) probes and two specific primers, were used. All samples were genotyped in duplicate.

### 2.5. Statistical Analyses

Since distributions of most of the analyzed quantitative parameters were significantly different from the normal distribution (Shapiro–Wilk test), we used the non-parametric Mann–Whitney U-test to compare them between groups. Logistic regression conducted with JAMOVI was used to compare qualitative variables (pain threshold and pain tolerance) between genotype categories and between combat athletes and the control group [22]. A chi-squared test was used to test the Hardy–Weinberg equilibrium. *p* < 0.05 was considered statistically significant.

## 3. Results

The genotype frequencies of the *SCN9A* rs6746030 polymorphism did not differ from the Hardy–Weinberg expectations for both athletes (GG—77.6%, GA—20.6%, AA—1.8%, chi-squared = 0.29, *p* = 0.86) and controls (GG—75.0%, GA—23.9%, AA—1.1%, chi-squared = 0.27, *p* = 0.87).

The results of anthropometric measurements are presented in Table 1. The examined athletes were older and shorter compared to the students. They did not differ significantly in body mass. Body mass index (BMI) indicated the normal weight of all study participants, and the differences between groups were mainly due to differences in body height.

There were no significant differences between the *SCN9A* genotype distributions and pain perception, defined as pain threshold and pain tolerance, in the group of combat athletes. However, significant differences in the control group were found. The odds ratio of having decreased pain tolerance (PPT2 > 10.00 kg/cm^2^) for the GA and AA genotypes was more than three times higher (3.11, CI: 95% 1.17 to 8.25, *p* = 0.02) than for the GG genotype homozygotes (Table 2).

**Figure 1:**
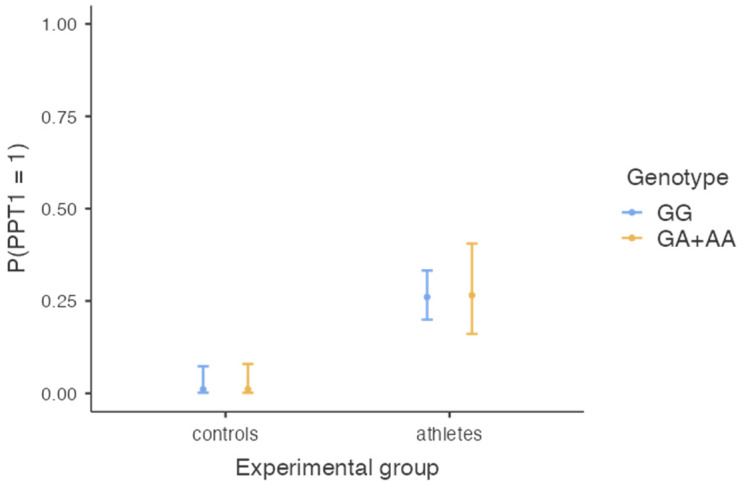
The chance of having high pain threshold (PPT1 > 10.0 kg/cm^2^) as a function of the *SCN9A* genotype and experimental group.

In Table 2, we present the proportion of combat athletes and controls with different *SCN9A* genotypes who demonstrated high and low pain threshold as well as pain tolerance. The model of binominal logistic regression with the experimental group (combat athletes vs. controls) and genotype (GG vs. GA + AA) as predictors and pain threshold (PPT1) as a dependent variable was significant, χ^2^(2) = 37.19, *p* < 0.001, R^2^ Nagerkelke = 18.5%. The effect of the experimental group was significant, B = 3.47, SE = 1.02, Z = 3.41, *p* < 0.001, OR = 32.25, 95%CI (4.39, 236.92), meaning that the probability of having a high pain threshold was higher in the combat athletes’ group (probability = 0.26, SE = 0.04, 95%CI (0.20, 0.34)) than in controls (probability = 0.01, SE = 0.01, 95%CI = (0.002, 0.07)). The effect of the genotype was not significant, B = 0.03, SE = 0.37, Z = 0.07, *p* = 0.944, OR = 1.03, 95%CI (0.50, 2.11) (see also Figure 1). Adding an interaction between the two predictors did not improve the model significantly, χ^2^(1) = 2.84, *p* = 0.092, meaning that the difference in pain threshold between athletes and controls was independent of their genotype.

**Figure 2:**
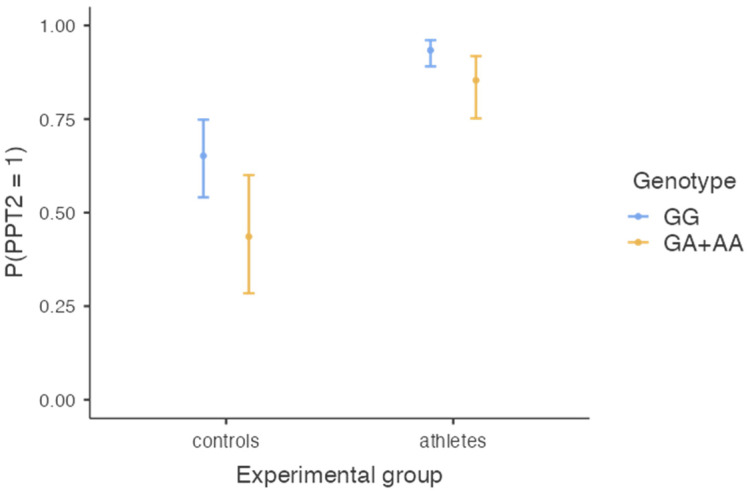
The chance of having high pain tolerance (PPT2 > 10.0 kg/cm^2^) as a function of the *SCN9A* genotype and experimental group.

The model of binominal logistic regression with the experimental group (combat athletes vs. controls) and genotype (GG vs. GA + AA) as predictors and pain tolerance (PPT2) as a dependent variable was significant, χ^2^(2) = 46.55, *p* < 0.001, R^2^ Nagerkelke = 23.2%. The effect of the experimental group was significant (B = 2.02, SE = 0.331, Z = 6.10, *p* < 0.001, OR = 7.57, 95%CI (3.94, 14.45)), meaning that the probability of having high pain tolerance was higher in the combat athletes’ group (probability = 0.90, SE = 0.02, 95%CI (0.85, 0.94)) than in controls (probability = 0.55, SE = 0.06, 95%CI = (0.43, 0.66)). The effect of the genotype was also significant (B = −0.88, SE = 0.36, Z = −2.45, *p* = 0.014, OR = 0.41, 95% CI (0.20, 0.84)), meaning that those with GA and AA genotypes were less likely to have high pain tolerance (probability = 0.68, SE = 0.07, 95%CI (0.54, 0.79)) than those with the GG genotype (probability = 0.84, SE = 0.03, 95%CI (0.78, 0.88)) (see also Figure 2). Adding an interaction between the two predictors did not improve the model significantly, χ^2^(1) = 0.54, *p* = 0.463, meaning that the difference in pain tolerance between athletes and controls was independent of their genotype, and the difference between genotypes was independent of whether the participant was a combat athlete or not.

## 4. Discussion

The aim of our study was to determine (1) whether the proportion of participants with low pain sensitivity (PPT1 and PPT2) was higher in combat athletes than in men who do not play sports professionally and (2) whether the probability of low pain sensitivity (PPT1 and PPT2) was higher in the group of participants with the GG genotype than in the participants with AA and GA genotypes. The results showed that the probability of having increased pain threshold and pain tolerance was higher in the group of combat athletes than in the control group, and thus we confirmed the first hypothesis. Our results are consistent with the literature, which states that physically active people, including athletes, have higher pain tolerance [2,3,5,17]. This variable may be modulated by numerous factors, such as the systematic exposure of the body of athletes to short-term high-intensity stimuli during training sessions, more frequent injuries, and significantly more frequent stimulation of the nervous system compared to non-athletes. From this perspective, the intensity of physical activity appears to be inextricably linked to pain perception. For athletes, pain is an integral part of their lifestyle. Many hours of training and participation in competitions, in this case, strenuous fights, classify combat athletes in particular as a group exposed to high pain intensity [5]. Consequently, many studies have confirmed that combat athletes have a higher pain tolerance than active controls and non-combat athletes [23,24]. Participants in a sport that requires contact face the additional challenge of being intentionally injured by other players. This may present an additional external mechanical stimulus and would activate pain pathways associated with skin and muscle deformation that may or may not be associated with tissue damage, rather than those that are natural in the muscle during exercise. The combination of these experiences might indeed lead to altered pain perception [5,24,25], as we have shown in this study.

To our knowledge, the present study is the second study to investigate the association between the rs6746030 polymorphism in the *SCN9A* gene and pain perception in combat athletes and people with low physical activity. The *SCN9A* gene encodes the NaV1.7 α-subunit isoform of the sodium channel. Ion channels, which are responsible for cell excitability and thus intercellular communication, play an important role in the biological mechanisms that generate and maintain neuropathic pain. Of the three voltage-gated channels (sodium, calcium, and potassium channels) directly involved in regulating membrane potentials, sodium channels appear to be the most important targets for pain [7]. Two genome-wide association studies (GWAS) performed in primary erythromelalgia patients in 2004 and 2005 revealed gain-of-function mutations in the *SCN9A* gene [26,27]. This association was confirmed by Nassar et al. [28] who knocked out of the *Scn9a* gene in mice. They showed that the mutants had reduced thermal and mechanical sensitivity under initial and inflammatory conditions [28]. Next, Cox et al. [13] sequenced chromosomal region 2q24.3, containing the *SCN9A* gene, from three consanguineous families with congenital insensitivity to pain. Sequence analysis revealed that channelopathy in *SCN9A* is a critical mediator of nociceptive pain [21]. Given the confirmed association between the *SCN9A* polymorphism rs6746030 and alterations in pain signaling in humans, this genetic variant has been extensively studied in several human genetic pain syndromes [15,16,29]. However, its effect on pain perception in athletes is almost unknown. Our study showed that the difference in pain threshold between athletes and control subjects was not related to the *SCN9A* genotype, but the effect of genotype on pain tolerance was significant. Specifically, all participants in the experimental group with genotypes GA and AA had lower pain tolerance than those with genotype GG. The obtained results are not in agreement with our previous study performed on 101 male boxers and 332 control participants, in which no association between the *SCN9A* rs6746030 polymorphism and phenotypic pain variables was found [30]. This confirms that the genetic background of pain is very complex and that we are only at the beginning of understanding the role of *SCN9A* variations in the athletes’ sensitivity to pain.

The results obtained in the present study support previous findings suggesting that carriers of the genotypes GA and AA have lower pain tolerance. The explanation for this could be as follows. The substitution of G to A in exon 18 of *SCN9A* results in a switch from positively charged arginine (R) to nonpolar tryptophan (W) at residue 1150 [31]. This substitution is located in a highly conserved sequence of the C-terminus of L2, the loop connecting the II and III domains of the Chan core. Considering the high conservation of the residue of the encoded protein from different species and the presence of a positively charged residue at this site in almost all other sodium channels, Estacion et al. [14] suggested that the substitution by the tryptophan residue has a functional impact on the biophysical properties of the NaV1.7 channel, which is known to play a key role in pain perception. Current-clamp analysis showed that the A (1150 W) allele depolarizes the resting membrane potential (6 mV) and increases the firing frequency twofold in response to depolarization in neurons of the spinal ganglion [14]. This observation was confirmed by Reimann et al. [15] who described a significant association between rs6746030 and pain score in patients with osteoarthritis, sciatica, phantom limb pain, lumbar discectomy, and pancreatitis, as well as in healthy women characterized by their responses to a variety of different noxious stimuli. Finally, the genotypes GA and AA were found to be associated with increased NaV1.7 activity and consequently altered pain threshold [14,15].

Current evidence, confirmed by the results of scientific research, including ours, shows that other factors such as physical activity significantly increases the pain threshold and pain tolerance, and that this effect is independent of the effect of the *SCN9A* genotypes. Thus, it appears that the effect of genotype on pain sensitivity can be modified by environmental factors such as exercise. Both pain threshold and pain tolerance need not be constant values for a given individual but can be modified by among others the environment. Additionally, it needs to be highlighted that pain phenotype is a polygenic trait. To date, about 252 genes have been linked to pain-associated behaviors [7]. It is more likely that several gene loci, each with a small but significant contribution, are responsible for this genetic component. Indeed, single-marker analysis is likely to make only a limited, but still important, contribution to understanding the pain perception.

## 5. Limitation

We are aware that our study has some limitations. Objective pain analyses are problematic because they are subjective and it is difficult to develop reliable scales to characterize pain. Moreover, in terms of the high emotional variability of individuals, it seems almost impossible to assess the quantitative and qualitative nature of pain. The weak point of the study was the algometer used to measure sensitivity to pain, which allowed the measurement to a value of 10.1 kg/cm^2^. In the case of the genetic association study, the relatively small group sample was the most important limitation. We studied a relatively small sample of Polish combat athletes. However, this group had an advantage—it included the best Poles with a uniform athletic level. Therefore, it is necessary to validate our results in larger cohorts of athletes as well as of different ethnicities.

## 6. Conclusions

The results of our study indicate that

- the combat athletes have increased pain threshold and pain tolerance;
- the *SCN9A* rs6746030 polymorphism may affect pain perception, in particular
- the GA and AA genotypes may be associated with lower pain tolerance compared with the GG genotype;
- the other factors, such as regular physical activity, significantly increases the pain threshold and pain tolerance, and this effect is independent of the effect of the *SCN9A* genotypes.

These results support the hypothesis that pain phenotype is a complex trait, important for achieving success in combat sports. Although the *SCN9A* rs6746030 polymorphism seems to be an important genetic marker for pain perception in athletes, more replication studies are needed.

## Figures and Tables

**Table 1:** Demographic and anthropometric data of the combat athletes and control group.

Variables	Combat Athletes (*n* = 214) Mean ± SD	Control Group (*n* = 92) Mean ± SD	*p*-Value
Age (years)	24.67 ± 6.57	21.21 ± 1.82	0.008
Height (cm)	178.6 ± 7.04	182.53 ± 8.04	<0.001
Body mass (kg)	78.21 ± 13.15	78.25 ± 10.19	0.956
BMI (kg/m^2^)	24.46 ± 3.33	23.43 ± 2.06	0.050

*p*-values evaluated by means of the Mann–Whitney U-test. Mean and standard deviations are given.

**Table 2:** The *SCN9A* genotype in relation to PPT measurements in combat athletes and control group.

Pain Threshold and Pain Tolerance		Combat Athletes (*n* = 214)		Control Group (*n* = 92)	
		GG	GA + AA	GG	GA + AA
PPT1 ≤ 10.0 kg/cm^2^	*n*	122	36	69	22
	%	73.5 %	75.0%	100.0%	95.7%
PPT1 > 10.0 kg/cm^2^	*n*	44	12	0	1
	%	26.5 %	25.0%	0.0%	4.3%
PPT2 ≤ 10.0 kg/cm^2^	*n*	12	6	23	14
	%	7.2 %	12.5%	33.3%	60.9%
PPT2 > 10.0 kg/cm^2^	*n*	154	42	46	9
	%	92.8 %	87.5%	66.7%	39.1%

PPT1—pain threshold; PPT2—pain tolerance.

## Data Availability

The data presented in this study are available on request from the corresponding author. The data are not publicly available due to privacy/ethical restrictions.
